# INTEGRATING DIVERSE CANCER PERSPECTIVES: FROM DEVELOPMENT TO IMPLEMENTATION

**DOI:** 10.1093/geroni/igac059.932

**Published:** 2022-12-20

**Authors:** Sean Halpin

## Abstract

Cancer among older adults is pervasive, putting excessive strain at the individual, caregivers, and wider society levels. In our symposium, we bring together researchers from varied disciplines—with a focus on easing the strain of cancer on older adults by identifying important gaps in care and developing and implementing innovative methods for improving health services. First, Carrion will discuss the multifactorial experience of fears and beliefs about cancer and cancer prevention in 57 older Latino men. Krok-Schoen will discuss the longitudinal association between religiosity and cognitive functioning among older adults with hematological cancers. Next, Seaman will discuss engaging hard-to-reach patients and those who underutilize the health system. Blackberry will then describe an implementation and impact framework of a five-year research program to improve care in older people with cancer. Lastly, Halpin will discuss the 36-month implementation of a video-based patient education program for patients with multiple myeloma who are preparing for autologous stem cell transplant. Understanding the development and implementation of programs aimed at improving health services among older adults with cancer will help improve understanding potential methods for identifying and addressing health services challenges in these populations.

